# Knock-Down of the IFR1 Protein Perturbs the Homeostasis of Reactive Electrophile Species and Boosts Photosynthetic Hydrogen Production in *Chlamydomonas reinhardtii*

**DOI:** 10.3389/fpls.2017.01347

**Published:** 2017-08-03

**Authors:** Deepak Venkanna, Christian Südfeld, Thomas Baier, Sarah V. Homburg, Anant V. Patel, Lutz Wobbe, Olaf Kruse

**Affiliations:** ^1^Faculty of Biology, Center for Biotechnology (CeBiTec), Bielefeld University Bielefeld, Germany; ^2^Faculty of Engineering and Mathematics, Fermentation and Formulation of Biologicals and Chemicals, Bielefeld University of Applied Sciences Bielefeld, Germany

**Keywords:** *Chlamydomonas reinhardtii*, photobiological hydrogen production, isoflavone reductase-like proteins, short-chain dehydrogenases/reductases, reactive electrophile species, singlet oxygen response 1 (*sor1*)

## Abstract

The protein superfamily of short-chain dehydrogenases/reductases (SDR), including members of the atypical type (aSDR), covers a huge range of catalyzed reactions and *in vivo* substrates. This superfamily also comprises isoflavone reductase-like (IRL) proteins, which are aSDRs highly homologous to isoflavone reductases from leguminous plants. The molecular function of IRLs in non-leguminous plants and green microalgae has not been identified as yet, but several lines of evidence point at their implication in reactive oxygen species homeostasis. The *Chlamydomonas reinhardtii* IRL protein IFR1 was identified in a previous study, analyzing the transcriptomic changes occurring during the acclimation to sulfur deprivation and anaerobiosis, a condition that triggers photobiological hydrogen production in this microalgae. Accumulation of the cytosolic IFR1 protein is induced by sulfur limitation as well as by the exposure of *C. reinhardtii* cells to reactive electrophile species (RES) such as reactive carbonyls. The latter has not been described for IRL proteins before. Over-accumulation of IFR1 in the singlet oxygen response 1 (*sor1*) mutant together with the presence of an electrophile response element, known to be required for SOR1-dependent gene activation as a response to RES, in the promoter of *IFR1*, indicate that IFR1 expression is controlled by the SOR1-dependent pathway. An implication of IFR1 into RES homeostasis, is further implied by a knock-down of *IFR1*, which results in a diminished tolerance toward RES. Intriguingly, *IFR1* knock-down has a positive effect on photosystem II (PSII) stability under sulfur-deprived conditions used to trigger photobiological hydrogen production, by reducing PSII-dependent oxygen evolution, in *C. reinhardtii*. Reduced PSII photoinhibition in *IFR1* knock-down strains prolongs the hydrogen production phase resulting in an almost doubled final hydrogen yield compared to the parental strain. Finally, *IFR1* knock-down could be successfully used to further increase hydrogen yields of the high hydrogen-producing mutant *stm6*, demonstrating that *IFR1* is a promising target for genetic engineering approaches aiming at an increased hydrogen production capacity of *C. reinhardtii* cells.

## Introduction

Among the most urgent challenges of our society today, are those associated to global warming, depletion of fossil fuels and a steady increase of the energy demand, which can pose a threat to economic and political stability (Organisation for Economic Co-operation and Development [OECD]/International Energy Agency [IEA], 2011). Photosynthesis-driven H_2_ production by photosynthetic microbes, such as cyanobacteria and microalgae, has a perfect carbon footprint, because of its zero CO_2_ emission. Within photobiological hydrogen production electrons and protons from water splitting are directed via photosynthesis toward specific H_2_-evolving enzymes, the hydrogenases (Gaffron and Rubin, 1942). Microalgae exploit Fe–Fe hydrogenases, which compared to other hydrogenases are highly efficient because of their extraordinarily high turnover number (Volgusheva et al., 2013; Lubitz et al., 2014). However, due to its oxygen sensitivity (Ghirardi et al., 1997), oxygenic photosynthesis cannot be directly coupled to hydrogen production in green microalgae. Therefore, photobiological hydrogen production has to be split into a two-stage process, which can be achieved by the experimental protocol proposed by Melis et al. (2000). This protocol relies on biomass generation under sulfur-replete conditions in the first stage and subsequent withdrawal of sulfur to trigger photoinhibition of photosystem II, resulting in a continuous decline of photosynthetic oxygen evolution, while mitochondrial respiration remains relatively unaffected by the lack of sulfur in the medium. In sealed culture flasks, this cultivation regime helps establishing anaerobic conditions, which are a prerequisite for the induction of the hydrogenase pathway (Ghysels and Franck, 2010). In *Chlamydomonas reinhardtii*, sulfur deprivation results in a strong down-regulation of the Calvin cycle and photosynthetic light reactions, based on a rapid decrease of Rubisco levels (Zhang et al., 2002) and an impaired PSII repair cycle, which relies on the *de novo* synthesis of the PSII subunit D1, which is restricted by the limited availability of sulfur-containing amino acids under these conditions (Wykoff et al., 1998). Although a massive decline in water-splitting activity is a prerequisite for the establishment of anaerobic conditions, which enable hydrogen production via the oxygen-sensitive hydrogenase enzyme, several studies clearly demonstrated that residual PSII activity and linear electron transport toward the hydrogenase are indispensable for efficient hydrogen production in *C. reinhardtii* (Antal et al., 2003; Volgusheva et al., 2013; Baltz et al., 2014; Steinbeck et al., 2015). The *C. reinhardtii* mutant *stm6* (Schönfeld et al., 2004) displays an enhanced hydrogen production capacity (Kruse et al., 2005) and its increased rate of mitochondrial oxygen consumption (Uhmeyer et al., 2017), was proposed to protect PSII during sulfur deprivation by accelerating the establishment of anaerobic conditions (Volgusheva et al., 2013), where irreversible, oxygen-dependent photoinhibition (Vass et al., 1992) cannot occur. Besides the PSII-dependent pathway of hydrogen production, starch degradation and subsequent glycolysis can provide NADH, which can be used to feed electrons into the photosynthetic electron transport chain without the need for water-splitting at PSII (Chochois et al., 2009; Baltz et al., 2014). Therefore, larger starch reserves present in *stm6* compared to wild type strains also contribute to the higher hydrogen production capacity seen for this mutant (Kruse et al., 2005; Doebbe et al., 2010). Cyclic electron flow (CEF) around photosystem I competes with electron delivery to the hydrogenase and a reduced CEF activity of *stm6* is another important aspect of its phenotype, which should significantly contribute to the elevated hydrogen production potential (Kruse et al., 2005). In addition to its photobiological production, hydrogen can also be produced under dark fermentative conditions in *C. reinhardtii* (Grossman et al., 2011).

With the aim to generate *C. reinhardtii* strains producing increased amounts of hydrogen upon exposure to sulfur limitation, several strategies have already been applied, which mainly targeted the oxygen sensitivity of the hydrogenase, the competition between CEF and hydrogen production, the efficiency of light conversion in the antenna and cellular starch contents (for review see Dubini and Ghirardi, 2015). Transcriptomics conducted with *C. reinhardtii* cells subjected to hydrogen production conditions could be another strategy to identify novel gene targets for the optimization of hydrogen production via genetic engineering (Nguyen et al., 2011; Toepel et al., 2013).

In a previous study (Nguyen et al., 2011), a transcript encoding the protein IFR1 (Cre11.g477200; NmrA-like) accumulated strongly in hydrogen-producing cells of *C. reinhardtii*. NmrA-like proteins belong to the protein superfamily of atypical short-chain dehydrogenases/reductases (aSDRs), which also contains isoflavone reductase-like (IRL) proteins (Moummou et al., 2012). IRL proteins from higher plants such as OsIRL from rice, were proposed to be implicated in ROS homeostasis, as OsIRL expression is induced by ROS and an overexpression confers enhanced ROS tolerance (Kim et al., 2010). SDRs and aSDRs including IRL proteins remain poorly characterized in microalgae thus far (Moummou et al., 2012). Therefore, we analyzed the function of IFR1 by applying a forward genetics strategy based on the use of artificial microRNA (amiRNA)-mediated knock-down of IFR1 in two distinct *C. reinhardtii* strains and subsequent analysis of the resulting phenotype, with a special focus on photosynthetic hydrogen production.

## Materials and Methods

### Chemicals

3-(3,4-Dichlorophenyl)-1,1-dimethylurea (DCMU), 2,5-Dibromo-6-isopropyl-3-methyl-1,4-Benzoquinone (DBMIB), 2E-Hexenal, Hydrogen peroxide (H_2_O_2_), Methyl Viologen (MV), Neutral red (NR), and Rose Bengal (RB) were purchased from Sigma-Aldrich.

### Strains and Growth Conditions

*Chlamydomonas reinhardtii* wild type CC124 (137c mt-), 4A+ (137c background) and mutant CC4604- sor1 (mt+) (Fischer et al., 2012) were obtained from the Chlamydomonas Center. UVM4, a UV mutant derived from CC4350 (cw15 arg7-8 mt+) known to efficiently express nuclear transgenes (Neupert et al., 2009) was kindly provided by R. Bock (MPI for Molecular Plant Physiology, Potsdam-Golm). The MOC1 knock-out mutant *stm6* was generated via random insertion of plasmid pArg7.8 (Debuchy et al., 1989), carrying the *Arg7* gene, into the nuclear genome of the arginine auxotrophic strain, *CC1618*. The *MOC1*-complemented strain *B13* (Schönfeld et al., 2004) was generated by co-transforming *stm6* with a 37-kb *Moc1*-containing cosmid isolated from a cosmid library and the *Cry1* gene as a dominant selectable marker conferring resistance to emetine (plasmid p613; Nelson et al., 1994). All strains were grown photoheterotrophically in TAP (tris acetate phosphate) medium (Harris, 1989) at 25°C with continuous white light of 100 μmol m^-2^ s^-1^. Experiments were performed by using the cells from mid-log phase. For hydrogen production, cells were harvested and washed three times with TAP-S medium. The cells were finally suspended in TAP-S to the tune of ∼25 μg/ml of chlorophyll. Hydrogen setup and gas measurement was carried out as described previously (Doebbe et al., 2010). After 20 h of anaerobic conditions, the effect of DCMU on H_2_ production was assessed by adding 20 μM DCMU to the sealed bioreactors. Quantitative analysis of RES and ROS stress tolerance was evaluated by growing 2 × 10^6^ cells/ml in TAP at 100 μmol m^-2^ s^-1^ with following chemicals: 5 μM DBMIB, 500 μM 2*E*-Hexenal, 4 μM RB, 15 μM NR, 0.5 μM MV and 7 mM H_2_O_2_. Cell growth was determined by analyzing OD_680_ and cell count (Z2 cell and particle counter, Beckman Coulter) at 0 and 24 h and 10 μl of culture was spotted on TAP agar plate for recovery.

### Generation of IRL Knock-Down Strains

The artificial microRNA sequence for generating IRL knock-down was designed with a web based tool WMD3^1^. The amiRNA sequences were generated to target exons 2 (*forward*: ctagtCAGGTCCAGGAGATTGATATAtctcgctgatcggcaccatg ggggtggtggtgatcagcgcaTATAACAATCTCCTGGACCTGg; reverse: ctagcCAGGTCCAG GAGATTGTTATAtagcgctgatcaccaccacccccatggtgccgatcagcgagaTATATCAATCTCCTGGACCTGa) and 4 (forward: ctagtGAGCACGCTATTAAGGTCGTAtctcgctgatcggcaccatgg gggtggtggtgatcagcgctaTACGGTCTTA-ATAGCGTGCTCg and *reverse*: ctagcGAGCACGC TATTAAGACCGTAtagcgctgatcaccaccacccccatggtgccgatcagcgagaTACGACCTTAATAG GTGCTCa) of the coding region and cloned into vector pChlamiRNAi3int (Molnar et al., 2009). CC124 was transformed by electroporation (Jaeger et al., 2017) and *stm6* was transformed via glass beads as mentioned previously (Kindle, 1990). Transformants were selected on paromomycin (10 μg/ml) TAP agar plates and transferred to sulfur depleted medium for screening.

### Antibody and Recombinant Protein Production

The polyclonal antiserum directed against a 17 aa polypeptide IFR1 was raised in rabbit (Agrisera, Sweden). To heterologously express IFR1 in *Escherichia coli*, a codon optimized full length *IFR1* coding sequence (phytozome: Transcript Cre11.g477200.t1.2) was synthesized *de novo* (Genscript, United States) and cloned between the *Nde*I and *Xho*I restriction sites of expression vector pET-24a(+) (Novagen), enabling streptag-based affinity purification.

### RNA Extraction and Quantitative Real Time PCR

Real-time RT-PCR was performed with DNaseI (RQ1 RNase-free DNase, Promega)-digested total RNA samples which was subjected to reverse transcription and PCR amplification using the SensiFAST^TM^ SYBR No-ROX One-Step Kit (BIOLINE, Germany). SYBR Green I fluorescence was recorded on a DNA Engine Opticon (Bio-RAD, Germany). Per sample 100 ng total RNA were used and *RPL13* (Gene ID: 5718254) as well as RACK1 (GeneID: 5723548) served as housekeeping genes. The following primers were used within the study: *IFR1* (5′-ATGGCGACTAAGAAGCACAC-3′ and 5′-CGAAGCCTGCTCATTGTAGT-3′), *RPL13* (5′-ATTCTTGCCGGGCAGCAGATTGTG-3′ and 5′-TTGCGCAGGAAG CGGTCATACTTC-3′) and *RACK1* (5′-TCAACATCACCAGCAAGAAGG-3′ and 5′-CTGGGCATTTACAGGGAGTG-3′). Relative mRNA expression levels were calculated according to Pfaffl (Pfaffl, 2001).

### SDS-PAGE and Immunoblotting

Cells were pelleted (3000 × g, 3 min) and suspended in lysis buffer (60 mM Tris pH 6.8, 2% SDS, 10% glycerol and freshly added 1 mM Pefabloc). Total proteins were extracted via freeze-thaw cycle in liquid N_2_ and quantified by Lowry assay (BioRAD). The proteins were separated by a 12% Tris-glycine SDS-PAGE and blotted on to a nitrocellulose membrane. After overnight blocking (5% Milk powder in TBST with 0.1% Tween), the membrane was incubated at room temperature for 1.5 h with IFR1-specific antiserum (1:2500), washed and then incubated for 1 h with a peroxidase-conjugated anti-rabbit antibody (Agrisera, Sweden) for chemiluminescence detection (ECL: GE Healthcare). Signals were visualized using the FUSION-FX7 detection system (Peqlab, Germany). Protein bands were quantified with MyImageAnalysis software (ThermoFisher Scientific).

### Chlorophyll Fluorescence Analyses

To determine the maximum quantum yield (F_v_/F_m_), 2 ml samples of a culture were incubated in the dark and aerated for 20 min. Chlorophyll fluorescence changes were recorded during a 10 min induction curve with actinic light (800 μmol photons m^-2^ s^-1^) using a Mini PAM (Waltz) and F_v_/F_m_ calculated according to the following equation (Maxwell and Johnson, 2000):

$$\begin{array}{c} \frac{F_{v}}{F_{m}}\;=\;\frac{F_{m}-F_{0}}{F_{m}} \end{array}$$

### Construction of Fusion Protein and Confocal Microscopy

The IFR1 coding sequence, codon-optimized for the nuclear codon bias of *C. reinhardtii* was cloned into vector pOpt-mVenus_Paro (Lauersen et al., 2015) by using the *Nde*I::*Bgl*II and *EcoR*V::*EcoR*I restriction sites to obtain C-terminal and N-terminal fusions, respectively. Fluorescence imaging was accomplished with a confocal laser scanning microscope (LSM780, Carl Zeiss GmbH, Germany) with specific filters for chlorophyll and mVenus as described before (Lauersen et al., 2015).

### Statistical Analysis

The significance of results was evaluated with a student’s two-tailed *t*-test for independent samples. The significance threshold was set between *p* < 0.05 to *p* < 0.1. Error bars represent standard error (SE) and in case of box plots the whiskers represent variability within the first and third quartile.

## Results

### IFR1 Is an Atypical Short-Chain Dehydrogenase that Accumulates in the Cytosol of *C. reinhardtii* as a Response to Abiotic Stress

A previous study (Nguyen et al., 2011), demonstrated that a transcript encoding a putative isoflavone reductase (IFR1) accumulated significantly in hydrogen-producing cells of *C. reinhardtii*. An NCBI-BLAST search using the amino acid sequence of IFR1 (Phytozome locus name Cre11.g477200; *C. reinhardtii* v5.5) revealed that this protein contains a conserved phenylcoumaran benzylic ether reductase (PCBER) like domain (specific hit/e-value 1.55e^-68^). PCBERs are NADPH-dependent aromatic alcohol reductases, and are described as atypical members of the short-chain dehydrogenase/reductase (SDR) family (Min et al., 2003). Atypical SDRs possess an N-terminus characteristic of NAD(P)-binding proteins and a small C-terminal domain presumed to be involved in substrate binding (Filling et al., 2002; Persson et al., 2003; Kavanagh et al., 2008). In contrast to classical SDRs, they do not have the conserved active site tyrosine residue typically found in SDRs and contain an atypical glycine-rich NADP-binding motif reading GXGXXG or G[GA]XGXXG (Supplementary Figure S1). The amino acid sequence of IFR1 shows identities to other members of the protein family in the range of 20–30% (Supplementary Table S1), with the highest similarity found for isoflavone reductases (Babiychuk et al., 1995) and IRL proteins (Petrucco et al., 1996) (Supplementary Figure S1 and Table S1).

**FIGURE 1:**
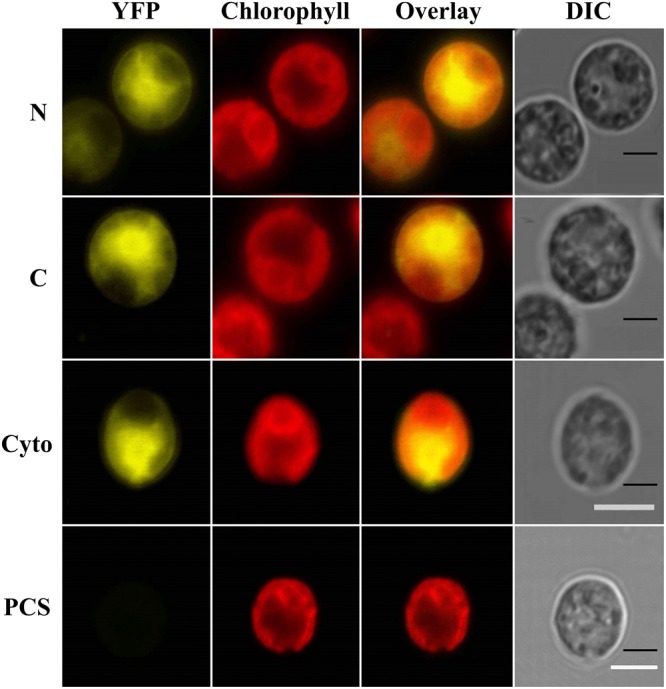
IFR1 localizes to the cytosol in *Chlamydomonas reinhardtii* cells. Laser scanning confocal microscopy detection of subcellular localization of the mVenus (yellow) fluorescent reporter fused to N- or C-terminus of IFR1 (N/C). A cell line expressing mVenus in the cytosol (Cyto, Lauersen et al., 2015) and the parental strain (PCS) served as controls. Individual imaging channels are presented, YFP: mVenus reporter signal in the yellow range, Chlorophyll: autofluorescence of chlorophyll visualized in the red range and used to orient the cells, Overlay: YFP and Chloro channel overlay, DIC: differential interference contrast. Scale bars represent 5 μm.

The *C. reinhardtii IFR1* gene encodes a 32 kDa protein whose localization was predicted to be cytosolic by the *in silico* prediction tool PredAlgo (Tardif et al., 2012). To confirm that IFR1 indeed resides in the cytosol, IFR1 was C- and N- terminally fused with YFP (mVenus variant; Kremers et al., 2006) and expressed in the *C. reinhardtii* cell line UVM4 (Neupert et al., 2009). Two strains, stably expressing either full length IFR1-YFP (C) or IFR1-YFP (N) were identified via immunoblots (Supplementary Figure S2; C and N). YFP fluorescence could be detected in both strains expressing YFP, either N- or C- terminally fused to IFR1 (**Figure 1**; N and C), while the parental control strain (PCS) only emitted red chlorophyll auto-fluorescence from the cup-shaped structure representing the chloroplast. Superimposition of the chlorophyll and YFP fluorescence demonstrated that the YFP-tagged IFR1 variants displayed a distribution of the YFP signal identical to that observed in the control strain (Cyto), expressing YFP in the cytosol (Lauersen et al., 2015). YFP-tagging of IFR1 in conjunction with confocal laser-scanning microscopy demonstrated that the localization of IFR1 is indeed cytosolic.

**FIGURE 2:**
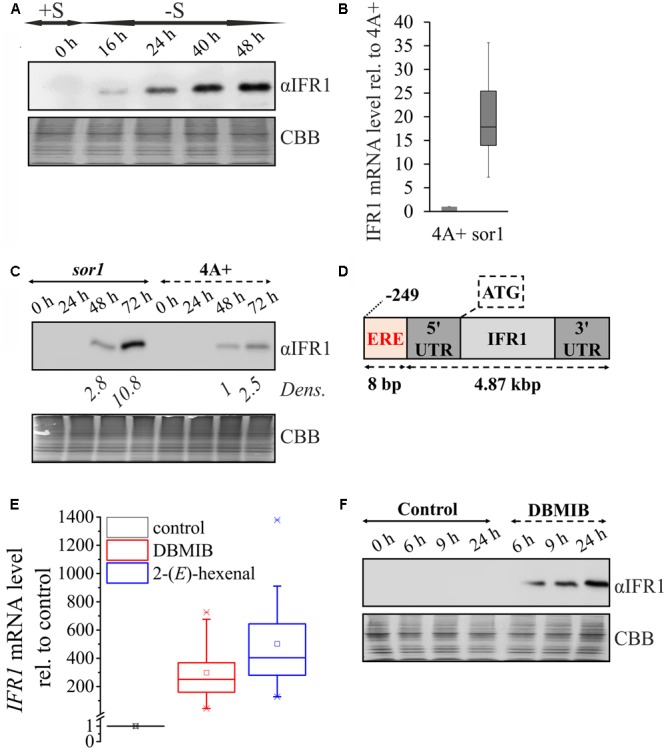
IFR1 accumulation is triggered by the SOR1-dependent pathway. **(A)** Samples were taken before (0 h, +S) and during the course of hydrogen production induced by sulfur deprivation of a wild type cell line (16–48 h; –S). IFR1 accumulation was analyzed with an IFR1-specific antiserum (αIFR1) and equal protein loading confirmed by colloidal Coomassie staining (CBB). **(B)** Comparison of *IFR1* mRNA levels in the *sor1* mutant (Fischer et al., 2012) and its parental strain (4A+) with samples taken in the late exponential phase. mRNA levels were determined by RTqPCR and the *IFR1* transcript level in 4A+ was set to 1. Median and interquartile range shown in the box-and-whisker diagram are derived from two biological replicates, each including nine technical replicates (*n* = 18). **(C)** Representative immunoblot (αIFR1) showing IFR1 accumulation during growth of mutant *sor1* and its parental strain (4A+) in nutrient-replete TAP medium for 3 days. Relative band intensities (*Dens.*) determined by densitometric scanning of immunblot signals are given relative to the band intensity of the 4A+ sample at t_48 h_ (set to 1). **(D)** Position of the octanucleotide motif CAACGTTG described to represent an electrophile response element (ERE; Fischer et al., 2012) implicated in the genetic response to reactive electrophile species (RES) and SOR1-dependent signaling relative to the start codon (ATG) of the 4.87 kbp IFR1 gene, comprising exons, introns and untranslated regions (UTRs). **(E)** *IFR1* mRNA levels determined by RTqPCR following dark treatment of WT cell cultures with DBMIB (5 μM) and 2-(*E*)-hexenal (500 μM) for 24 h. The mRNA level of the solvent control sample was set to 1. Median and interquartile range shown in the box-and-whisker diagram are derived from two biological replicates, each including six technical replicates (*n* = 12). **(F)** Immunoblot (αIFR1) showing IFR1 accumulation distinct time points (6–24 h) after the addition of DBMIB (5 μM) or only solvent (Control) to a liquid TAP culture of the *C. reinhardtii* wild type CC124 and subsequent dark incubation for 24 h.

In a previous study (Nguyen et al., 2011) we compared the transcriptomes of the *C. reinhardtii* wild type (wt) cc406 and the high hydrogen production mutant *stm6glc4* (Doebbe et al., 2007) [derived from *stm6* (Kruse et al., 2005)] during photosynthetic hydrogen production triggered by sulfur deprivation (Melis et al., 2000). The transcriptome data revealed that within the peak hydrogen production phase, *IFR1* transcripts accumulated to a high extent (∼10–40-fold compared to sulfur-replete condition) in both strains. In order to confirm that the accumulation of IFR1 transcript is indeed translated into elevated protein amounts, we analyzed protein samples taken at distinct points from a hydrogen-producing culture of a *C. reinhardtii* wild type (**Figure 2A**). A strong induction of IFR1 protein expression was observed in the wild type under S-deprived anaerobic H_2_ production conditions. IFR1 accumulation started before the onset of anaerobiosis and H_2_ production conditions (from 24 h onward), indicating that sulfur deprivation rather than anaerobiosis is required for IFR1 induction. An inspection of published RNAseq data sets using AlgaePath (Zheng et al., 2014) revealed that sulfur deprivation alone triggers *IFR1* mRNA accumulation [∼8-fold induction 6 h after withdrawal of sulfur; González-Ballester et al., 2010; gene expression omnibus (GEO) series GSE17970]. An even stronger induction was observed within a transcriptome study analyzing the modulation of the *C. reinhardtii* transcriptome in response to nitrogen depletion (∼46-fold after 48 h; Miller et al., 2010; GSE24367), but IFR1 protein expression could not be detected under nitrogen-deplete conditions (data not shown), demonstrating that IFR1 accumulation is not generally observed as a response to macronutrient limitation. While effects of carbon dioxide limitation on *IFR1* transcript accumulation were comparably small (∼2-fold; Fang et al., 2012; GSE33927), exposure of wild type *C. reinhardtii* cells to hydrogen peroxide led to a rapid accumulation of IFR1 transcript (∼19-fold within 1 h; Blaby et al., 2015; GSE34826). In addition, a previous transcriptome study indicated that *IFR1* belongs to the set of genes overexpressed in the mutant *singlet oxygen resistant 1* (*sor1*), which shows a constitutively higher expression of genes implicated in the detoxification of reactive oxygen and electrophile species [9.8 in *sor1* vs. 0 in parental (4A+); Fischer et al., 2012; GSE33548]. Overexpression of IFR1 mRNA in *sor1* vs. its parental strain (4A+) could be confirmed by RTqPCR experiments [median 17.8; lower quartile (Q1) 13.9; upper quartile (Q3) 25.4; IFR1 mRNA level in 4A+ set to 1; **Figure 2B**]. The higher mRNA level was also translated into higher IFR1 protein amounts found in *sor1* (**Figure 2C**; 48 and 72 h; sor1 vs. 4A+). Analysis of the IFR1 promoter region led to the identification of an 8 bp palindromic motif (CAACGTTG) (**Figure 2D**) which was identified as an electrophile response element (ERE) in nuclear promoters of *C. reinhardtii* genes overexpressed in the mutant *sor1* and whose expression is activated by reactive electrophile species (RES) (Fischer et al., 2012).

Indeed, treatment of *C. reinhardtii* WT cultures with the RES-compounds DBMIB (2,5-Dibromo-6-isopropyl-3-methyl-1,4-benzoquinone) and 2-(*E*)-hexenal triggered a strong accumulation of *IFR1* mRNA [median fold-induction vs. control: 250.5 for DBMIB and 403.5 for 2-(*E*)-hexenal; **Figure 2E**]. In contrast to DBMIB, 2-(*E*)-hexenal is a RES (oxylipin) that occurs naturally in high light-stressed cells of *C. reinhardtii* (Roach et al., 2017) and is formed from polyunsaturated fatty acids via peroxidation and subsequent enzymatic cleavage (Mosblech et al., 2009). Addition of DBMIB to sulfur-replete cultures of a *C. reinhardtii* WT in the dark induced a strong accumulation of IFR1 protein (**Figure 2F**). However, IFR1 protein expression could not be observed (data not shown) in TAP grown cultures supplemented with DCMU [3-(3,4-Dichlorophenyl)-1,1-dimethylurea; PSII forward electron inhibitor; Metz et al., 1986], indicating that inhibition of photosynthetic electron transport by DCMU or DBMIB can be excluded and noted effects can be mainly attributed to DBMIB’s action as a reactive electrophile.

### A Knock-Down of IFR1 Causes Diminished RES-Tolerance

**FIGURE 3:**
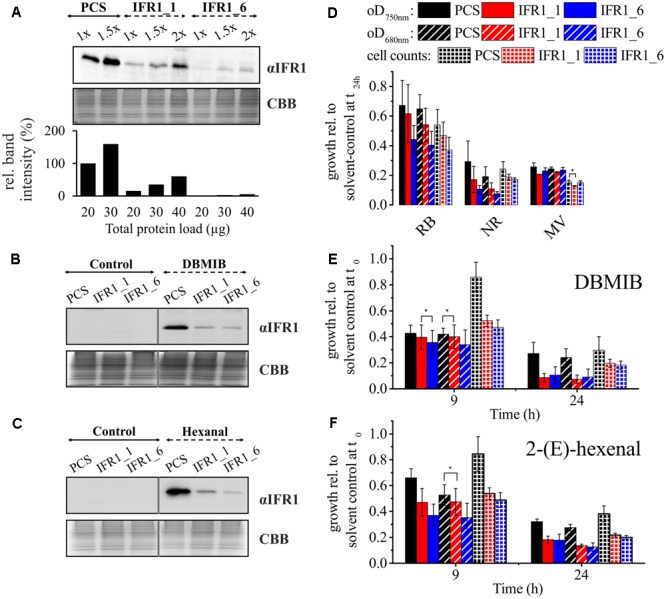
IFR1 knock-down causes diminished tolerance toward RES in *C. reinhardtii.* **(A)** Immunodetection of IFR1 protein (αIFR1) in the parental strain (PCS; wild type CC124) and IFR1 knock-down strains (IFR1_1 and IFR1_6) detected after 48 h of cultivation in sulfur deplete medium. A colloidal Coomassie stained gel (CBB) served as loading control. Different amounts of proteins were used and band intensities (lower bar diagram) determined by densitometric analysis (1x PCS set to 100%). **(B,C)** IFR1 accumulation in PCS and IFR1 knock-down strains grown for 24 h in TAP supplemented with DBMIB (5 μM) or 2-(E)-hexenal (500 μM). **(D)** Growth inhibition by reactive oxygen species determined for the PCS and the two IFR1 knock-down strains during 24 h of growth in TAP supplemented with 4 μM rose Bengal (RB), 15 μM neutral red (NR), or 0.5 μM methyl viologen (MV). Optical densities (determined at 680 and 750 nm) and cell counts are given relative to the untreated/solvent-control sample (set to 1). Error bars indicate standard errors derived from three biological replicates including technical replicates (*n* = 3). Asterisks indicate significant differences between PCS and knock-down strains according to a two-tailed Student’s *t*-test (*p* < 0.05). **(E,F)** Growth inhibition following treatment of PCS and IFR1 knock-down strains with 5 μM DBMIB and 500 μM 2-(E)-hexenal for 9 or 24 h in TAP medium. Standard errors are derived from three biological replicates, including technical replicates (*n* = 3). Except for the data indicated by asterisks (*p* > 0.05) differences between PCS and knock-down strains were significant according to a two-tailed Student’s *t*-test (*p* < 0.05).

To functionally characterize IFR1 of *C. reinhardtii*, we applied a reverse genetics approach, employing a nuclear expression vector for the expression of artificial microRNAs (amiRNA) (Molnar et al., 2009). Screening of transformants based on immunoblots with the IFR1-specific antiserum led to the identification of two knock-down strains. When grown in S-deplete medium, IFR1_1 and IFR1_6 accumulated ∼65 and ∼95% less IFR1 protein, respectively as compared to the parental strain (**Figure 3A**).

Diminished accumulation of IFR1 in both knock-down strains was also observed, when cells were treated with the RES compound DBMIB (**Figure 3B**). In line with the strong *IFR1* transcript accumulation observed after treatment with 2-(*E*)-hexenal (**Figure 2E**), addition of this compound to liquid cultures triggered a strong accumulation of IFR1 protein in the parental strain, which was diminished in knock-down strains (**Figure 3C**). Prompted by the finding that IFR1 protein accumulates following the exposure of *C. reinhardtii* cells to RES and the reported requirement of IRL proteins for oxidative stress tolerance in higher plants (Babiychuk et al., 1995; Kim et al., 2010), we analyzed the tolerance of IFR1 knock-down strains toward various compounds which either act as reactive oxygen/electrophile species (ROS/RES) or induce their cellular accumulation (**Figures 3D–F**). To this end, compounds inducing ROS-stress [rose bengal (RB); neutral red (NR), and methyl viologen (MV)] or acting as RES [DBMIB and 2-(*E*)-hexenal] were added to the cultures and the growth retarding-effect was quantified after 24 h via measurement of optical densities (oD_680 nm/750 nm_) and cell densities based on cell counting. The cultures were also spotted on TAP agar plates for recovery (Supplementary Figure S3). Significant differences reflected by all growth parameters applied could not be observed regarding the susceptibility of knock-down strains vs. parental strain toward methyl viologen, which triggers superoxide formation *in vivo* (Babbs et al., 1989) (**Figure 3D**; MV). Although neutral red and rose bengal, which act as photosensitizers and trigger the formation of singlet oxygen in live cells (Fischer et al., 2004), exerted a greater growth-inhibiting effect on both knock-down strains (**Figure 3D**), differences between the parental and IFR1 knock-down strains were not statistically significant according to a two-tailed Student’s *t*-test (*p* < 0.05). In contrast, statistically robust (*p* < 0.05; two-tailed Student’s *t*-test) differences could be seen when cells were treated for 24 h with DBMIB or 2-(*E*)-hexenal (**Figures 3E,F**), which caused a more pronounced growth inhibition in the knock-down strains. At least when cell counts were used as a growth parameter, a significantly higher susceptibility of knock-down strains toward RES could already be seen 9 h after the addition of DBMIB or 2-(*E*)-hexenal. A diminished availability of IFR1 in *C. reinhardtii* therefore reduces the tolerance toward RES.

### Prolonged Hydrogen Production by IFR1 Knock-Down Mutants

**FIGURE 4:**
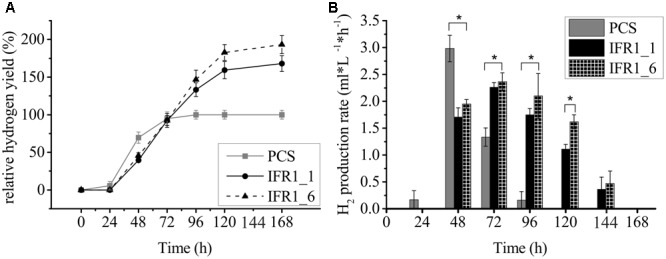
Prolonged hydrogen production in IFR1 knock-down strains compared to the wild type. **(A)** Time course of hydrogen production for the parental strain (PCS) and IFR1 knock-down strains. Hydrogen yields in the knock-down strains are given relative to the final yield of the parental strain (set to 100%). Each data curve represents an average of three biological replicates including three technical triplicates (*n* = 9) with error bars representing the standard error. **(B)** H_2_ production rates during the course of hydrogen production. Error bars indicate the standard error (*n* = 9) and asterisks indicate differences between PCS and knock-down strains which are significant according to a two-tailed Student’s *t*-test (^∗^*p* < 0.05).

IFR1 protein accumulates strongly in hydrogen producing cultures following sulfur-depletion (**Figure 2A**), indicating a potential role of this protein during the acclimation to sulfur depletion or anaerobiosis. The effect of *IFR1* knock-down was assessed by measuring H_2_ production of the knock-down strains (**Figure 4A**). Parental strain, IFR1_1 and IFR1_6 were grown in sulfur-replete TAP medium to a mid-log phase and transferred to sulfur-deplete TAP medium by adjusting them to the same starting chlorophyll concentration (∼25 μg/ml). Hydrogen production was first notable 48 h after the onset of sulfur depletion and at the beginning hydrogen yields in the PCS exceeded those of the knock-down strains by ∼35–40%. During the course of H_2_ production, production rates declined in the PCS from 48 h onward, while rates in the knock-down strains increased toward t_72 h_ and started declining notably beyond the time point t_120 h_ (**Figure 4B**). The H_2_ production phase in PCS ceased at 96 h with a production phase (time between the first detection and the end of H_2_ production) of 3 days as compared to 5 days by *IFR1* knock-down strains. Although the highest rate of hydrogen production (2.98 ± 0.25 ml L^-1^ h^-1^) was reached in the PCS strain, the prolonged hydrogen production in the knock-down strains eventually led to final hydrogen yields that were about 68 ± 10% (SE) (IFR1-1) and 93 ± 12% (IFR1-6) higher than the yield from the wild type.

### Prolonged H_2_ Production in IFR1 Knock-Down Strains Results from a Sustained PSII Activity

**FIGURE 5:**
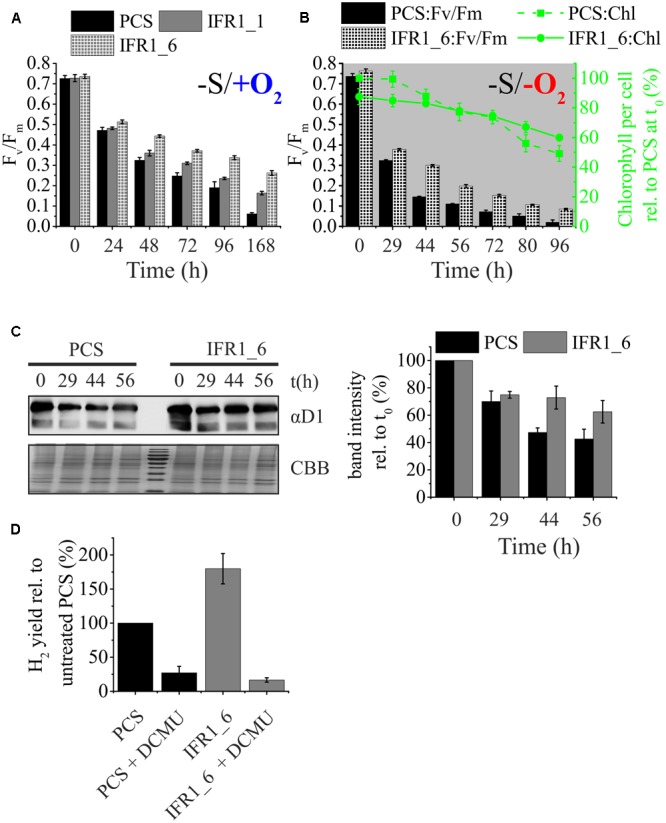
Contribution of PSII and photosynthetic/respiration (P/R) rates on hydrogen production. **(A)** Maximum quantum yield (F_v_/F_m_) of dark-adapted cells of the parental strain (PCS) and IFR1 knock-down strains (IFR1_1/IFR1_6) before (t_0_) and after exposure to sulfur limitation (t_24_–t_168 h_) and aerobic conditions. Error bars indicate the standard error from three biological replicates (*n* = 3). **(B)** Time course of the maximum quantum yield (F_v_/F_m_; left y-axis) and the cellular chlorophyll content (right y-axis) during photosynthetic hydrogen production of the parental strain (PCS) and one of the IFR1 knock-down strains (IFR1_6). Chlorophyll data were normalized to the chlorophyll content of PCS at t_0_ (set to 100%). Standard errors derived from three biological replicates (*n* = 3) are indicated as error bars. Except for t_0_, the differences between PCS and IFR1_6 in regard to F_v_/F_m_ were significant according to a two-tailed Student’s *t*-test (*p* < 0.05). **(C)** Representative immunoblot showing the immunodetection of PSII subunit D1 (upper left panel; αD1) in samples of the parental strain (PCS) and IFR1_6 taken at indicated times during a hydrogen production experiment. A colloidal Coomassie stain (lower left panel; CBB) served as a loading control. Results from densitometric scanning (right panel) of blot signals are given relative to the D1 signal intensity determined for t_0_ (set to 100%). Error bars indicate standard errors (three biological replicates; *n* = 3). **(D)** Relative H_2_ yields obtained with the parental control strain (PCS) (black bars) and knock-down strain IFR1_6 (gray bars) in the absence or presence of 20 μM DCMU. Hydrogen yields determined for the untreated PCS were set to 100%. Error bars represent standard error (*n* = 6).

One of the reasons for a prolonged hydrogen production in the course of sulfur starvation, could be a high residual PSII activity, which is required for efficient hydrogen production (Volgusheva et al., 2013; Steinbeck et al., 2015). Indeed, both IFR1 knock-down strains displayed a higher residual activity of PSII, measured as the maximum quantum yield of dark-adapted cells (F_v_/F_m_), following the exposure to sulfur limitation under aerobic conditions (**Figure 5A**; -S/+O_2_; Fv/Fm; t_72-168 h_). The knock-down strain IFR1_6 was then selected for more detailed analyses regarding differences in PSII stability between knock-down and parental strain under hydrogen production conditions (sulfur deprivation under anaerobic conditions). Also during the course of hydrogen production, the knock-down of IFR1 causes an increased stability of PSII, as seen by higher F_v_/F_m_ values from time point t_29 h_ onward (**Figure 5B**; -S/-O_2_; left y-axis; PCS vs. IFR1_6). The lowered susceptibility of PSII toward photoinhibition in the IFR1_6 was also reflected by a lower relative decrease in the cellular chlorophyll content (∼30% vs. 50% in PCS; **Figure 5B**; right y-axis; green curves). In good agreement with the higher residual PSII activity found in IFR1 knock-down strains (**Figures 5A,B**), the cellular content of the PSII core subunit D1 declined more slowly within the course of hydrogen production in knock-down strain IFR1_6 compared to its parental strain (**Figure 5C**).

Specific inhibition of PSII with DCMU was used to confirm if the electrons for prolonged H_2_ production indeed originated from residual PSII activity. DCMU was added directly into the H_2_ bioreactors 30 h after the onset of sulfur deprivation. DCMU blocks the PSII-dependent pathway of hydrogen production based on residual water-splitting activity and linear electron transport toward the hydrogenase enzyme and inhibits H_2_ production substantially, as reported before (Kruse et al., 2005; Volgusheva et al., 2007; Scoma et al., 2014). H_2_ production dropped in both strains upon addition of DCMU (**Figure 5D**), but the relative effect of DCMU on hydrogen production was much stronger in the IFR1 knock-down strain (73% reduction in PCS vs. 163% reduction in IFR1_6). Furthermore total hydrogen production in strain IFR1_6 (16.6 ± 3.2%) was lower than the production observed for the parental strain (27.1 ± 9.4%), when DCMU was added to inhibit PSII. It can thus be concluded that the increased hydrogen production capacity caused by a knock-down of IFR1 mainly results from an enhanced activity of the PSII-dependent pathway, especially during the later stages of the hydrogen production pathway, when PSII activity in IFR1 knock-down strains exceeds the respective activity seen in the parental strain (**Figures 5A–C**).

### IFR1 Knock-Down Can Be Applied as a Tool to Further Enhance Hydrogen Production in a Strain with a High Starting Capacity

**FIGURE 6:**
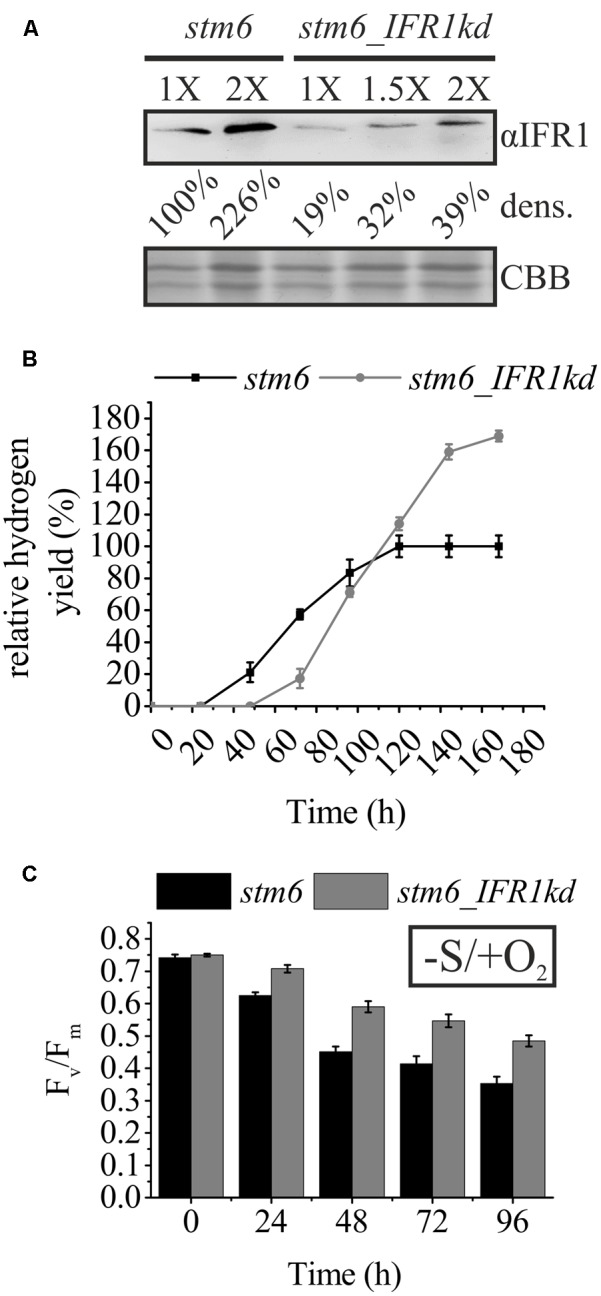
Knock-down of IFR1 in the boosts hydrogen production in the high hydrogen producer mutant *stm6.* **(A)** Immunoblot analysis of IFR1 accumulation in *stm6* and *stm6_IFR1kd* cultivated under sulfur-limiting conditions. Different amounts of total protein (1X; 1.5X; and 2X) were used for immunodetection of IFR1 (αIFR1) with a colloidal Coomassie stain (CBB) serving as a loading control. Results from densitometric signal analysis (dens.) are indicated. **(B)** Relative time-dependent H_2_ yields of the *stm6* parental strain (black curve) and *stm6_IFR1kd* (gray curve) with the final yield in *stm6* set to 100%. Error bars represent the standard error (three biological replicates including technical triplicates, *n* = 9). **(C)** Maximum quantum yield of PSII determined after dark incubation (Fv/Fm) determined in cultures of *stm6* (black bars) and *stm6_IFR1kd* (gray bars) exposed to sulfur starvation. Standard errors, shown as error bars are derived from three biological replicates including technical duplicates (*n* = 6). Except for t_0_, differences between *stm6* and *stm6_IFR1kd* were significant according to a two-tailed Student’s *t*-test (*p* < 0.05).

To test whether the knock-down of *IFR1* can be applied as a tool to improve the hydrogen production capacity in various *C. reinhardtii* strains, we selected the strain *stm6*, known to produce high amounts of hydrogen (Kruse et al., 2005; Doebbe et al., 2010; Nguyen et al., 2011). One of the created *IFR1* knock-down strains, *stm6_IFR1kd*, displayed an IFR1 accumulation diminished to ∼20% of the IFR1 level found in the parental strain *stm6* (**Figure 6A**; αIFR1; 1X *stm6* vs. 1X *stm6_IFRkd*). Confirming the results obtained with knock-down strains derived from a wild type cell line (**Figure 4**), an IFR1 knock-down in the background of strain *stm6* also had a tremendous impact on the time course of hydrogen production and the overall production capacity (**Figure 6B**). The onset of hydrogen production in strain *stm6_IFRkd* (**Figure 6B**; gray curve) was delayed by ∼20 h compared to the parental strain (black curve), but hydrogen production in the knock-down strain reached a plateau phase only at time point 168 h, while the parental strain reached this phase already before t_120 h_. A prolonged hydrogen production phase together with an increased H_2_ productivity rate (up to 3.07 ml⋅ L^-1^⋅h^-1^ at t_72 h_), indicated by a steeper slope of the *stm6_IFRkd* curve, resulted in a final hydrogen yield of the knock-down strain which was 70% higher than the respective yield obtained with the parental strain.

In analogy to what has been observed for the *IFR1* knock-down strains derived from a wild type, PSII activity (F_v_/F_m_; **Figure 6C**) declined more slowly in *stm6_IFRkd* vs. *stm6* when cells were cultivated under aerobic sulfur-limiting conditions (0.48 + 0.01 in *stm6_IFRkd* vs. 0.35 + 0.02 in *stm6* at t_96 h_). In summary, these results demonstrate again that a diminished IFR1 level boosts hydrogen production in *C. reinhardtii* and that this effect is based on a sustained residual PSII activity which extends the hydrogen production phase significantly. The correlation between cellular amounts of IFR1 and hydrogen production capacity is further underscored by the diminished hydrogen yields obtained with the *sor1* mutant (Supplementary Figure S4), which overexpresses IFR1 (**Figures 2B,C**), in comparison to its parental strain (4A+).

## Discussion

*In silico* analyses performed with the amino acid sequence of IFR1 revealed that this protein represents an atypical member of the short-chain dehydrogenase/reductase (SDR) superfamily. Several SDRs including IFR1 from *C. reinhardtii* have been suggested to be referred to as NmrA-like family proteins (family designation SDR48A) according to a nomenclature initiative of Persson et al. (2009). Most of these SDRs including IFR1, however, share significantly higher percent identities with isoflavone reductases (*Arabidopsis thaliana* IFR; ∼29% identity to IFR1), IRL proteins (*Zea mays* IRL; ∼29%), phenylcoumaran benzylic ether reductases (PCBER; ∼28%) or eugenol synthases (EGS; ∼24%) from higher plants than with the N metabolite repression protein A (NmrA; ∼20%) from *Aspergillus (Emericella) nidulans* (Supplementary Figure S1 and Table S1). A wide-scale bioinformatics study on SDRs in plant genomes suggested a distinct SDR family for IFR, PCBER and eugenol synthase (family designation SDR460A) and although IFR1 was 1 of 15 *C. reinhardtii* proteins that could not be assigned to any SDR family during that study, a high homology of IFR1 to members of the SDR460A family was claimed, however (Moummou et al., 2012).

In the legume alfalfa (*Medicago sativa*), isoflavone reductase (IFR) catalyzes the stereospecific reduction of 2′-hydroxyformononetin to yield (3R)-vestitone (Dewick, 1977; Paiva et al., 1991) as part of the biosynthesis pathway for the isoflavonoid (-)-medicarpin (Guo and Paiva, 1995). Isoflavonoids and IFRs are almost entirely confined to legumes and, although flavonoids have been identified as sex pheromones in *Chlamydomonas eugamentos* (Birch et al., 1953), no isoflavonoids or IFRs have been reported in *C. reinhardtii* (May et al., 2008; Annamalai and Nallamuthu, 2014). Several IFR-like (IRL) proteins have been cloned from non-leguminous plants (Babiychuk et al., 1995; Petrucco et al., 1996; Shoji et al., 2002; Kim et al., 2003; Hua et al., 2013). Despite their high homology toward IFR proteins, IRL proteins do not accept 2′-hydroxyformononetin as a substrate (Petrucco et al., 1996) and for several higher plant IRLs their specific induction by abiotic stresses such as exposure to reactive oxygen species (Kim et al., 2010) or UV light (Lers et al., 1998) has been demonstrated. For the IRL proteins from maize and rice, a strong negative correlation between cellular glutathione (GSH) levels and the expression level of IRLs was shown (Petrucco et al., 1996). Sulfur depletion, which triggers the accumulation of maize IRL, is a condition known to cause a strong decline of GSH levels in green algae (Salbitani et al., 2015) and higher plants (Kopriva and Rennenberg, 2004). As observed within the present study, *C. reinhardtii* IFR1 also accumulates upon sulfur limitation, whereas its expression is low under stress-free conditions (**Figure 2A**).

In further analogy to IRL proteins from maize and rice, expression of the *C. reinhardtii IFR1* gene is also induced by reactive oxygen species such as hydrogen peroxide (Blaby et al., 2015). Molecular details on the expression regulation of higher plant *IRL* genes have thus far remained obscure, whereas for IFR1 the present study provides strong evidence for the function of an ERE (Fischer et al., 2012) as a *cis*-regulatory *IFR1* promoter sequence required for the accumulation of IFR1 following exposure to reactive electrophiles (**Figures 2B–F, 3C**). The ERE *cis*-regulatory element (CAACGTTG) was identified as a palindromic sequence overrepresented in the -70 to -340 bp promoter region of genes overexpressed in the *singlet oxygen resistant 1* (*sor1*) mutant and the ERE of *IFR1* lies within this region (-249 bp; **Figure 2D**). It was shown to be required for the induction and overexpression of ROS/RES-defense genes (glutathione-*S*-transferase 1; *GSTS1*) by the lipophilic RES-compound DBMIB in mutant *sor1*. Reporter constructs containing the ERE responded more strongly and much faster to lipophilic RES than to hydrophilic chemicals producing ROS. From this and other results it was therefore concluded that ERE activation via ROS is indirect and based on lipid peroxidation triggered by ROS and generating lipophilic RES such as malondialdehyde (Fischer et al., 2012). Among a variety of ROS- and RES-generating chemicals tested, *GSTS1* reporter constructs containing ERE elements responded most strongly to DBMIB and 2-(*E*)-hexenal (Fischer et al., 2012), and IFR1 transcript (**Figure 2E**) and protein (**Figures 2F, 3B,C**) accumulates upon treatment of *C. reinhardtii* cells with these compounds. Furthermore, IFR1 mRNA and protein over-accumulate in the *sor1* mutant (**Figures 2B,C**).

In addition to their strong effects on IFR1 expression, the growth-retarding effect of DBMIB and 2-(*E*)-hexenal is more prominent in IFR1 knock-down strains than in their parental strain (**Figures 3E,F**). It is therefore tempting to speculate, that the short-chain dehydrogenase/reductase (SDR) IFR1 might be involved in the detoxification of these compounds, since (SDRs) have already been shown to act as cytosolic aldehyde reductases (CytADRs) in *A. thaliana* (Yamauchi et al., 2011). In principle, highly reactive 2-alkenals from lipid peroxidation can be detoxified either by reduction of the aldehyde group or by reduction of the α, β-unsaturated bond (Mano et al., 2005; Yamauchi et al., 2011). CytADRs catalyze the latter reaction and represent typical SDRs while IFR1 is an atypical SDR (aSDR) according to its NAD(P)H binding motif of the G-X-X-G-X-X-G type (Supplementary Figure S1). Alignment of the IFR1 amino sequence with those of CytADRs from *A. thaliana* revealed percent identities in the range of 19–21%, whereas a *C. reinhardtii* SDR (Cre12.g549852) whose expression is induced by 2-(*E*)-hexenal (Fischer et al., 2012) shows a higher degree of identity to CytADRs (23–25% identity; Supplementary Table S1). In *A. thalinana*, enzymes reducing reactive carbonyls have been identified within the medium-chain dehydrogenase/reductase (MDR) superfamily, NADB_Rossmann (SDR) superfamily and aldo-keto reductase (AKR) superfamily (Yamauchi et al., 2011), but aSDRs implicated in the detoxification of reactive carbonyls have not been identified so far. *In vitro* assays based on NADPH consumption monitored via absorbance change at 340 nm with recombinant IFR1 and 2-(E)-hexenal (data not shown) did not indicate that this compound could represent a substrate *in vivo*.

Apart from the treatment of cell cultures with lipophilic RES, sulfur deprivation is a condition that is associated with IFR1 accumulation (**Figure 2A**). In microalgae sulfur limitation is known to cause the formation of reactive oxygen species (Salbitani et al., 2015), which could in turn trigger lipid peroxidation resulting in the production of reactive carbonyls/RES (Mosblech et al., 2009; Roach et al., 2017). At the same time, the withdrawal of sulfur diminishes the glutathione pool size (Salbitani et al., 2015) and a large pool of reduced glutathione (GSH) protects cellular components (e.g., amino groups from DNA bases or within proteins) against modification by RES via scavenging as GSH-conjugates and subsequent detoxification (Mueller and Berger, 2009). It is therefore possible that *IFR1* induction under sulfur-deplete conditions proceeds via the accumulation of RES, originating from -S-triggered ROS formation and simultaneous impairment of GSH-dependent scavenging, and an activation of the SOR1-dependent pathway. This could also provide an explanation for the finding that nitrogen limitation does not induce IFR1 accumulation, because at least in higher plants, effects of nitrogen deficiency on foliar GSH levels are rather small compared to those exerted by sulfur withdrawal (Koprivova et al., 2000).

Although IFR1 does not seem to be involved in the direct detoxification of RES, a diminished amount of IFR1 reduces RES tolerance in *C. reinhardtii* (**Figures 3E,F**), indicating that IFR1 is somehow implicated in the regulation of RES homeostasis. The precise *in vivo* function of IFR1 within the context of RES homeostasis in *C. reinhardtii* can, however, not be depicted at the moment. Since *in vivo* substrate identification based on *in silico* analyses of SDRs and aSDRs is not feasible, future research on IFR1 will have to comprise the cumbersome screening of compound libraries (Bhatia et al., 2015).

Intriguingly, a knock-down of IFR1 in *C. reinhardtii* boosts hydrogen production (**Figures 4, 6**). In *C. reinhardtii*, the production of hydrogen can be triggered by sulfur deprivation in air-tight cultures (Melis et al., 2000). A strong down-regulation of the Calvin–Benson cycle is thought to over-reduce the photosynthetic electron transport chain, a condition which promotes the formation of ROS. ROS damage the photosynthetic apparatus, especially photosystem II, and a diminished activity of the PSII repair cycle in the absence of sulfur further contributes to a strong decline in PSII activity, which in turn gradually decreases the oxygen content of cultures, because mitochondrial respiration is less affected by sulfur deprivation (Ghysels and Franck, 2010). A strong decline of PSII activity could also be noted under sulfur limitation in the present study (**Figures 5A,B, 6C**) and remarkable differences were seen between parental strains and IFR1 knock-down mutants. Interestingly, in strains expressing lower amounts of IFR1, PSII was less susceptible to -S-induced photoinhibition, as could be seen as higher F_v_/F_m_ values (**Figures 5A,B, 6C**) and a slower decline in the levels of D1 protein (**Figure 5C**), constituting the PSII core complex. The increased stability of PSII in knock-down strains caused a prolonged hydrogen production phase in strains with diminished IFR1 levels. Therefore, the data from the present study nicely underscore the importance of residual water-splitting activity for -S-induced hydrogen production, which was also observed in previous studies (Volgusheva et al., 2013).

The simultaneous occurrence of a higher RES sensitivity and increased stability of PSII as important phenotypic characteristics of *IFR1* knock-strains first seems counterintuitive. In this context, however, it must be emphasized that RES should not be merely viewed as cytotoxic compounds that need be rapidly removed from the cellular metabolism in order to prevent cell damage. Numerous studies indicate a role of RES as important signaling molecules which represent a central component of abiotic stress responses (Yamauchi et al., 2015; Muench et al., 2016). For instance, 2-(*E*)-hexenal has been shown to be a strong inducer of genetic programs activated as part of abiotic stress responses (Copolovici et al., 2012; Yamauchi et al., 2015) and this compound is formed under photooxidative stress conditions [e.g., as experienced by plants devoid of non-photochemical quenching mechanisms (Loreto et al., 2006)]. The PSII-damaging effect of 2-(*E*)-hexenal was found to be rather subtle in higher plants (Yamauchi et al., 2015). Therefore, the more pronounced growth retarding effect of 2-(*E*)-hexenal seen for *IFR1* knock-down strains might be based on mechanisms other than PSII inhibition. Overall, it seems feasible that perturbation of RES-dependent signaling could also result in a higher stress tolerance of cells (e.g., by a diminished threshold for the activation of stress response mechanisms or even their constitutive activation).

As a novel finding, manipulation of RES homeostasis in *C. reinhardtii* can be used to increase photosynthetic hydrogen production. Although the precise molecular function of IFR1 in *Chlamydomonas* is difficult to depict at the moment, the regulation of IFR1 expression via a characterized ERE, its strong over-accumulation in the *sor1* mutant and the lowered RES tolerance indicate that IFR1 is a factor required for RES-dependent signaling or RES handling in this microalga (**Figures 2B–D, 3E,F**). IFR1 will thus represent an important tool for future studies regarding the role of RES in abiotic stress responses of *C. reinhardtii*.

## Author Contributions

DV performed most of the experiments; DV, SH, CS, and TB performed and designed experiments; DV, LW, AP, and OK conceived the project and wrote the article with contributions of all the authors.

## Conflict of Interest Statement

The authors declare that the research was conducted in the absence of any commercial or financial relationships that could be construed as a potential conflict of interest.

## Footnotes

1 http://wmd3.weigelworld.org
