# Supplementary material for: Knock-Down of the IFR1 Protein Perturbs the Homeostasis of Reactive Electrophile Species and Boosts Photosynthetic Hydrogen Production in Chlamydomonas reinhardtii
Source: Front Plant Sci. 2017 Aug 3;8:1347. doi: 10.3389/fpls.2017.01347 (PMC5540887; doi:10.3389/fpls.2017.01347)
Supplement: Supplementary file 1 [file Table_1.DOCX]

Supplementary Material

Perturbation of reactive electrophile species homeostasis boosts photosynthetic hydrogen production in *Chlamydomonas reinhardtii*

Deepak Venkanna, Sara Vanessa Homburg, Christian Südfeld, Thomas Baier, Lutz Wobbe, Anant V. Patel & *Olaf Kruse

*** Correspondence:** Corresponding Author: olaf.kruse@uni-bielefeld.de

# Supplementary Figures and Tables

## Supplementary Figures


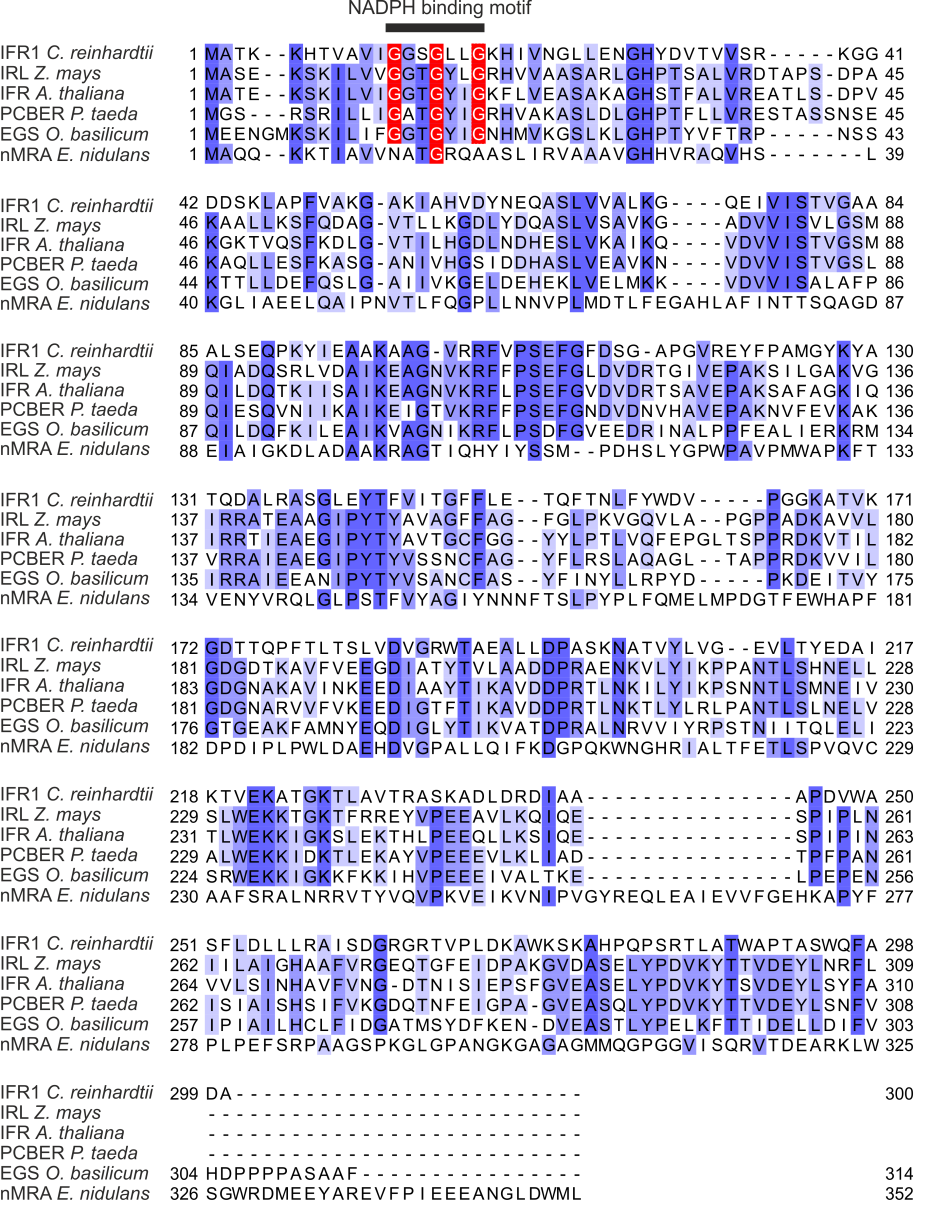


**Supplementary Figure 1.** Alignment of amino acid sequences of different atypical SDRs with the *C. reinhardtii* IFR1 amino acid sequence. Conserved glycine residues within the NADPH binding motif are highlighted in red, while other conserved amino acids are highlighted by light/dark blue color according to the degree of conservation. Aligned sequences: *Chlamydomonas reinhardtii* IFR1 (Phytozome locus name Cre11.g477200; *C. reinhardtii* v5.5); *Pinus taeda* PCBER (DDCBER1 UniProtKB O81651); *Zea mays* IRL (UniProtKB P52580); *Arabidopsis thaliana* IFR (UniProtKB P52577**)**, *Ocimum basilicum* EGS (UniProtKB A0A1B2U6R8) and *Emericella nidulans* NMRa (UniProtKB Q5AU62).


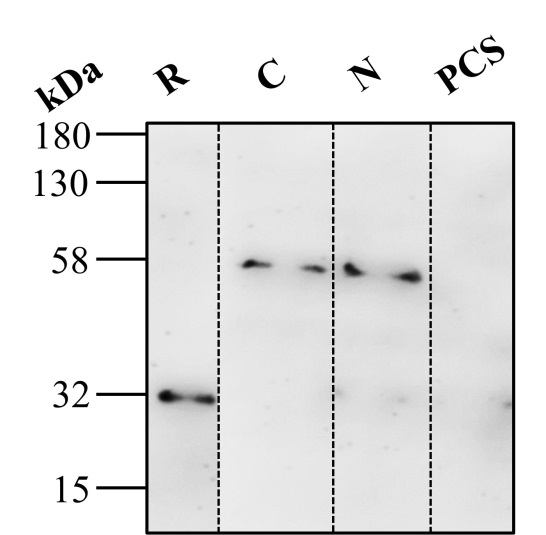


**Supplementary Figure 2.** Immunodetection of YFP-tagged IFR1 in cell lines generated for confocal laser-scanning microscopy. Immunodetection of IFR1 in whole cell extracts derived from the parental strain UVM4 (PCS) and transformants expressing N- and C-terminal fusions of IFR1 with YFP (N/C) and recombinant IFR1 (R, 32 kDa) using an antiserum raised against recombinant IFR1.


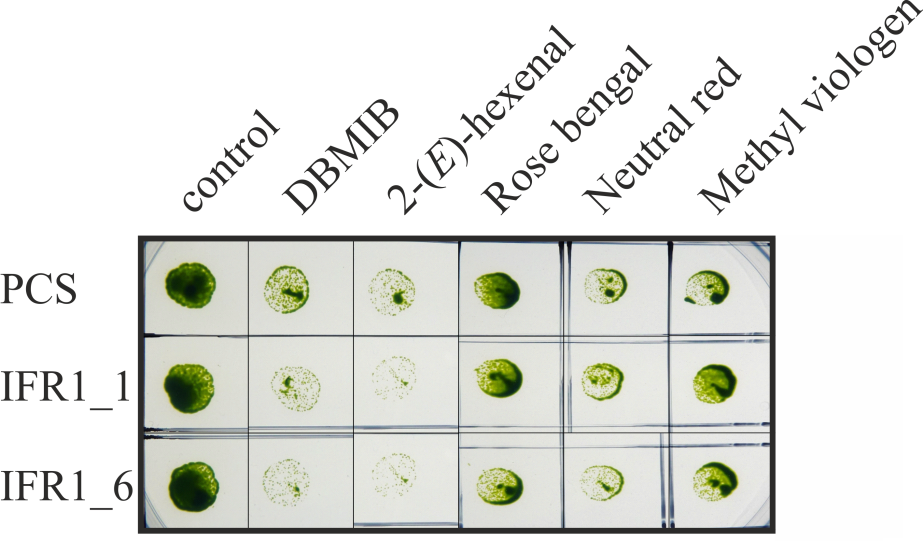


**Supplementary Figure 3.** Recovery of CC124 (PCS) and IFR1 knockdown mutants after growth analyses in the presence of various ROS-inducing chemicals (Rose bengal 4 µM, Neutral red 15 µM and Methyl viologen 0.5 µM) and RES (DBMIB 5 µM, 2-(*E*)-hexenal 500 µM) agents. Cells grown for 24 h in liquid TAP supplemented with defined concentrations of individual chemicals were spotted on TAP agar plates for recovery. Cells recovered over a period of 4 days in presence of white light (100 µmol m^-2^ s^-1^).


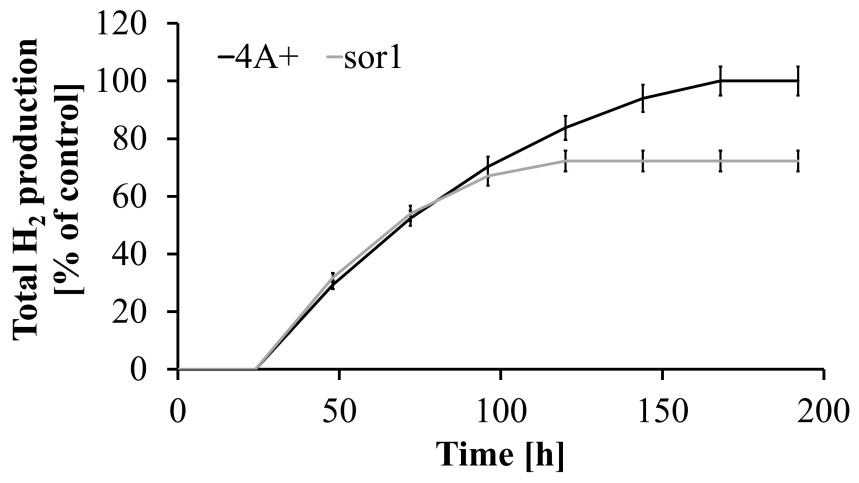


**Supplementary Figure 4.** Hydrogen production by the parental strain (4A+) is set to 100%. An average of two biological replicates with technical triplicates each represented. Error bars indicate the standard error.

## Supplementary Tables

| **Atypical SDR family member** | **EC number** | **Organism** | **UniProtKB accession number** | **Reference** | **% identity to IFR1 from *C. reinhardtii*** |
| --- | --- | --- | --- | --- | --- |
| Isoflavone reductase like protein  IRL |  | *Z. mays* | P52580 | Petrucco et al. 1996 | 28.8 |
| Isoflavone reductase  IFR | 1.3.1.45 | *A. thaliana* | P52577 | Babiychuk et al. 1995 | 28.6 |
| Eugenol synthases  EGS | 1.1.1.318 | *O. basilicum* | A0A1B2U6R8 | Louie et al. 2007 | 24.5 |
| NmrA-type negative transcriptional regulators  NMRA |  | *E. nidulans* | Q5AU62 | Andrianopoulos et al. 1998 | 19.8 |
| Phenylcoumaran benzylic ether reductase (PCBER)  DDCBER1 |  | *P. taeda* | O81651 | Min et al. 2003 | 27.6 |
| Cre12.g549852  SDR induced by hexenal-treatment |  | *C. reinhardtii* | A8JHG2 | Fischer et al., 2012 | 20.0 |
| At2g24190  (cytosolic aldehyde reductase (ADR)) |  | *A. thaliana* |  | Yamauchi et al., 2011 | 20.8  (23.5 to Cre12.g549852 |
| At3g61220  (cytosolic aldehyde reductase (ADR)) |  | *A. thaliana* |  | Yamauchi et al., 2011 | 19.1  (24.8 to Cre12.g549852 |
| At1g01800  (cytosolic aldehyde reductase (ADR)) |  | *A. thaliana* |  | Yamauchi et al., 2011 | 19.0  (23.0 to Cre12.g549852 |

**Supplementary Table 1.** Atypical short-chain dehydrogenase (SDR) members from various organisms and their UniProtKB accession numbers, available enzyme commission (E.C.) numbers, references and their amino acid sequence identity to IFR1 from *C. reinhardtii*.
